# Bile-Liver phenotype: Exploring the microbiota landscape in bile and intratumor of cholangiocarcinoma

**DOI:** 10.1016/j.csbj.2025.03.030

**Published:** 2025-03-18

**Authors:** Lei Wang, Hui Zhao, Fan Wu, Jiale Chen, Hanjie Xu, Wanwan Gong, Sijia Wen, Mengmeng Yang, Jiazeng Xia, Yu Chen, Daozhen Chen

**Affiliations:** a Institute for Reproductive Health and Genetic Diseases, The Affiliated Wuxi Maternity and Child Health Care Hospital of Nanjing Medical University, Wuxi 214002, China; b Department of Hepatopancreatobiliary Surgery, The Affiliated Wuxi No.2 People’s Hospital of Nanjing Medical University, Wuxi 214002, China; c Department of Hepatopancreatobiliary Surgery, Jiangnan University Medical Center, Wuxi 214002, China; d Department of Hospital Infection Management，Wujin Affiliated Hospital of Nanjing University of Traditional Chinese Medicine, Changzhou 213161, China; e Institute for Reproductive Health and Genetic Diseases, Wuxi Maternal and Child Health Hospital, Wuxi School of Medicine, Jiangnan University, Wuxi 214002, China; f Jiangsu Provincial Key Laboratory on Parasite and Vector Control Technology, Jiangsu Institute of Parasitic Diseases, Wuxi 214064, China; g Department of General Surgery, The Affiliated Wuxi No.2 People’s Hospital of Nanjing Medical University, Wuxi 214002, China; h Department of General Surgery, Jiangnan University Medical Center, Wuxi 214002, China

**Keywords:** Cholangiocarcinoma16S rRNA gene profiling, Microbiota, Biletype, Staphylococcus

## Abstract

Cholangiocarcinoma (CCA) arises within the peritumoral bile microenvironment, yet microbial translocation from bile to intracholangiocarcinoma (IntraCCA) tissues remains poorly understood. Previous studies on bile microbiota alterations from biliary benign disease (BBD) to CCA have yielded inconsistent results, highlighting the need for cross-study analysis. We presented a comprehensive analysis of five cohorts (N = 266), including our newly established 16S rRNA gene profiling (n = 42), to elucidate these microbiota transitions. The concordance of bacteria between CCA bile and intraCCA tissue, represented by Enterococcus and Staphylococcus, suggested microbiota migration from bile to intratumoral tissues. A computational random forest machine learning model effectively distinguished intraCCA tissue from CCA bile, identifying Rhodococcus and Ralstonia as diagnostically significant. The model also excelled in differentiating CCA bile from BBD bile, achieving an AUC value of 0.931 in external validation. Using unsupervised hierarchical clustering, we established Biletypes based on microbial signatures in our cohort. A combination of 17 genera effectively stratified patients into Biletype A and Biletype B. Biletype B robustly discerned CCA from BBD, with Sub-Biletype B1 correlating with advanced TNM stage and poorer prognosis. Among the 17 genera, bacterial Cluster 1, composed of Sphingomonas, Staphylococcus, Massilia, Paenibacillus, Porphyrobacter, Lawsonella, and Aerococcus, was enriched in Biletype B1 and predicted CCA with an AUC of 0.96. Staphylococcus emerged as a promising single-genus predictor for CCA diagnosis and staging. In conclusion, this study delineates a potential microbiota transition pathway from the gut through CCA bile to intra-CCA tissue, proposing Biletypes and Staphylococcus as biomarkers for CCA prognosis.

## Highlights

• Microbiota migrate from the gut through CCA bile to intra-CCA tissue.
• Machine learning models successfully differentiate intra-CCA tissue from CCA bile.
• Biletypes could predict the diagnosis, TNM stage, and prognosis of CCA.
• Staphylococcus serves as single-genus predictor for CCA diagnosis and staging.

## Graphical Abstract

**Figure:**
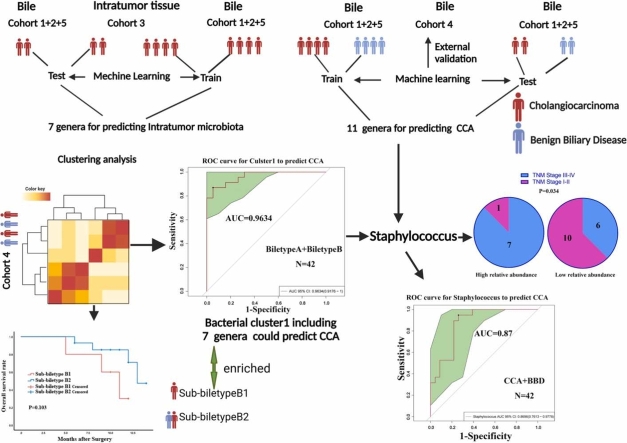

## 1 Introduction

Cholangiocarcinoma (CCA), a malignancy arising from both intrahepatic and extrahepatic bile ducts, has shown a concerning annual increase in incidence. Despite advances in oncology, the prognosis for CCA patients remains dismal, with limited therapeutic options available [1], [2]. Current chemotherapy regimens and targeted therapies show minimal efficacy, and the response to immunotherapy, particularly anti-PD-1/PD-L1 monoclonal antibodies, remains suboptimal [3].

The burgeoning field of gut microbiome research has revolutionized our understanding of disease pathogenesis, particularly in colorectal cancer [4], inflammatory bowel disease[5], and central nervous system disorders [6]. Fecal microbiota transplantation has emerged as a promising therapeutic strategy for these conditions [7], [8]. Of particular interest is the emerging role of specific bacterial species in modulating the tumor immune microenvironment. For instance, *Fusobacterium nucleatum* has been shown to confer immunotherapy resistance in colorectal cancer through its metabolite succinate [9]. In addition to colorectal cancer, numerous studies have focused on characterizing the gut microbiota in CCA patients, revealing distinct microbial signatures associated with disease progression [10], [11]. However, given the anatomical and functional continuity between the biliary system and gastrointestinal tract, little is known to date about the role of bile microbiota in the carcinogenesis of CCA [10]. Gut microbiota represented by *Fusobacterium nucleatum* may migrated to the bile to play a pivotal role in CCA pathogenesis. 16 s rRNA sequencing of bile samples has revealed Fusobacteria as a characteristic component of biliary microbiota in CCA patients [12]. Similarly, while gut *Staphylococcus* species have been implicated in colorectal cancer progression [13], the potential role of bile *Staphylococcus* in CCA warrants investigation. This study leverages bile 16 s sequencing data to establish the diagnostic, staging, and prognostic relevance of bile *Staphylococcus* species in CCA, evaluating their predictive accuracy.

The concept of microbial signatures, exemplified by enterotypes in colorectal cancer [14], [15] and hepatotypes in liver cancer [16], has provided valuable insights into tumor biology and patient outcomes. Building upon this paradigm, we have successfully established distinct biletypes based on microbial composition, demonstrating significant correlations with clinicopathological features and prognosis. These CCA-associated biletypes, characterized by specific microbial signatures, offer promising predictive potential for CCA diagnosis and management.

Despite significant progress in gut microbiota research, the characterization of cancer-associated bile microbiota remains largely unexplored. Previous investigations into the composition and temporal dynamics of bile microbiota in CCA patients have often produced conflicting results [17], [18], [19], [20]. To address these inconsistencies, our study introduces an innovative multi-cohort 16S rRNA gene profiling framework combined with machine learning algorithms to identify and validate microbiota signatures predictive of CCA progression. Our comprehensive analysis categorizes patients into five distinct groups: CCA bile, benign biliary disease (BBD) bile, intratumoral tissue (IntraCCA), paratumoral tissue (ParaCCA), and normal hepatic tissue (Normal). This stratification enables precise discrimination between BBD and CCA-associated bile microbiota, overcoming the limitations of previous studies through a more systematic and robust analytical approach.

Notably, previous researches revealed distinct compositional differences between bile microbiota and intratumoral microbiota. Saab et al. [20] previously identified *Enterococcus*, *Streptococcus*, *Bacteroides*, *Klebsiella*, and *Pyramidobacter* as dominant genera in CCA bile through 16S rRNA gene profiling of 28 CCA samples. Conversely, Chai et al. [21] reported *Burkholderiales* and *Pseudomonadales* as highly abundant bacteria in CCA tissue samples. Given that CCA originates within the biliary microenvironment, the potential migration of bile microbiota into CCA tissues and their subsequent impact on carcinogenesis remains unexplored. This study aims to elucidate the compositional differences between CCA bile and intra-CCA tissues, thereby clarifying the microbiota migration process from the biliary system to tumor tissue. Furthermore, we employ machine learning to develop a predictive model capable of identifying bacterial strains that differentiate intra-CCA tissue from CCA bile, providing novel insights into the microbial dynamics of CCA pathogenesis.

## 2 Methods

### 2.1 Cohort acquisition, inclusion criteria and sample collection

We systematically searched four major genomic databases—the DNA DataBank of Japan (DDBJ, https://www.ddbj.nig.ac.jp/), European Nucleotide Archive (ENA, http://www.ebi.ac.uk/ena), NCBI Sequence Read Archive (SRA, http://www.ncbi.nlm.nih.gov/sra), and Genome Sequence Archive (GSA, https://ngdc.cncb.ac.cn/gsa/)—using the search terms "cholangiocarcinoma," "bile duct cancer," "gallbladder cancer," "16S rRNA," and "metagenome," filtered by "Bioproject." Bioprojects containing high-throughput sequencing reads and associated metadata were compiled. Additionally, we extracted relevant articles from NCBI PubMed using the keywords "cholangiocarcinoma," "bile duct cancer," "gallbladder cancer," "microbiome," "16S rRNA," and "metagenome". The search cutoff date was set to February 22, 2025. Studies lacking complete sequencing information or including acute inflammatory cases were excluded. Three independent researchers, blinded to each other's assessments, reviewed each study. When raw data were unavailable publicly, corresponding authors were contacted to request the data.

Inclusion criteria for studies in this analysis were as follows: a) Samples derived from human bile, bile duct tissue, or gallbladder tissue; b) 16S rRNA gene or metagenome sequenced by next-generation sequencing; c) No record of antibiotic or chemotherapy use within three months prior to sampling; d) Availability of associated metadata and sequencing data from public databases or provided by authors via email by February 22, 2025.

Ultimately, 16S rRNA sequencing data and metadata from four studies [12], [19], [20], [21], were included, designated as Cohorts 1, 2, 3, and 4 for further analysis (Table 1). Raw FASTQ data were obtained from the DDBJ, ENA, and NCBI BioProject databases (accession numbers DRA011518, PRJEB43183, PRJNA753723, and PRJNA793871). Cohort 4 served as an external validation set. Fourteen additional studies were excluded due to incomplete sequence or metadata information. The low incidence of CCA poses a significant limitation to large-scale biliary tumor studies.

**Table 1:** Summary of studies on CCA microbiota-related information.

**Study**	**PMID**	**Sample type**	**Bioproject**	**number of cases**	**Platform**	**Sequencing**	**Variable Regions**	**Raw data**	**Inclusion study order**
Kirishima 2022	35624429	Human bile juice		244	Illumina	16 s rRNA	V3-V4	Unavailable	-
**Ito 2022**	**36358797**	**Human bile juice**	**DRA011518**	**18**	**Illumina**	**16 s rRNA**	**V3-V4**	**available**	**Cohort 1**
**Saab 2021**	**33690612**	**Human bile juice**	**PRJEB43183**	**75**	**Illumina**	**16 s rRNA**	**V3-V4**	**available**	**Cohort 2**
**Chai 2023**	**36563106**	**Human tissue**	**PRJNA753723**	**99**	**Illumina**	**16 s rRNA**	**V3-V4**	**available**	**Cohort 3**
Choi 2021	34282606	Human bile juice		25	Illumina	16 s rRNA	V3-V4	Unavailable	-
Song 2020	32526082	GBC and BBD tissue		14	Illumina	Metagenome	--	Unavailable	-
Tsuchiya 2018	29693356	Human bile juice		37	Illumina	16 s rRNA	V3-V4	Unavailable	-
Okuda 2022	35610303	saliva, gastric juice, pancreatic juice, bile, feces et al.		11	Mykinso® technology manufactured by Cykinso Inc	16 s rRNA	unavailable	Unavailable	-
Lee 2020	31980025	extracellular vesicles in the plasma		155	Illumina	16 s rRNA	V3-V4	Unavailable	-
Avilés-Jiménez 2016	26493848	Brushed Epithelial cells of human		20	Illumina	16 s rRNA	V4	Unavailable	-
Xin 2024	38461467	Human tissue		210	Illumina	16 s rRNA	V4	Unavailable	
Kim 2021	33876584	Human bile juice		24	Illumina	16 s rRNA	V3-V4	Unavailable	
Miyabe 2022	35565248	Human bile juice	**PRJNA793871**	43	Illumina	16 s rRNA	V3-V4	**available**	**Cohort 4**
Dangtakot 2021	33507704	Human bile juice		60	Illumina	16 s rRNA	V3-V4	Unavailable	
Zhang 2023	38030815	Human feces and bile juice		58	Illumina	16 s rRNA	V3-V4	Unavailable	
Poudel 2023	37071646	Human bile juice		46	Illumina	16 s rRNA	V3-V4	Unavailable	
Shukla 2024	38845533	Human bile juice		60	Illumina	16 s rRNA	V3-V4	Unavailable	
Chng 2016	27428430	Human tissue		122	Illumina	16 s rRNA	V3-V6	Annotation unavailable	

To address this limitation, we established Cohort 5, comprising 19 CCA patients (aged 42–84 years) treated at the Affiliated Wuxi No. 2 People’s Hospital of Nanjing Medical University between February 2022 and December 2023. A control group of 23 patients with BBD was selected from the same period. Controls were rigorously matched to CCA patients by gender, age, and underlying comorbidities (including hypertension, diabetes, coronary artery disease, chronic intestinal disease, and other malignancies) (P > 0.05). None of the participants had a history of antibiotic or chemotherapy use within three months prior to bile collection. Bile samples from the BBD group were aspirated from excised gallbladders, while CCA bile was collected aseptically from the gallbladder or via percutaneous transhepatic cholangial drainage (PTCD). All samples were immediately flash-frozen in liquid nitrogen and stored at −80°C until analysis.

### 2.2 Batch effect control

To control for batch effects, we implemented a standardized sample collection protocol across all cohorts [22]. To mitigate primer-induced noise in 16S datasets, primers targeting the V3–V4 regions were used [23]. All raw sequencing data were processed using the USEARCH-OTU pipeline, with taxonomic annotation performed against the SILVA database (Version 138.1) using 16S-specific parameters to ensure uniformity in data clustering and taxonomic assignment. Additionally, we employed rigorous quality control measures and advanced statistical methods, including permutational multivariate analysis of variance (PERMANOVA), to identify and account for potential batch effects[24], [25].

### 2.3 Sequence processing

Raw sequencing data from all cohorts were downloaded in FASTQ format. Meta-sequence processing across multiple cohorts was conducted following established protocols [26]. Strict quality control was implemented at each processing step using FASTP [27] (version 0.18.0). Representative OTU sequences were taxonomically classified using a naive Bayesian model based on the SILVA database [28] (version 138.1). Biomarker features for each group were identified using LEfSe software [29] (version 1.0). Within-sample (alpha) diversity metrics were calculated using QIIME [30] (version 1.9.1) and the picante package [31] (version 1.8.2). Between-sample (beta) diversity was assessed using Bray-Curtis distances, analyzed with the R Vegan package (version 2.5.3).

### 2.4 DNA extraction, PCR Amplification and Illumina Sequencing

DNA extraction was performed using a bacterial genomic DNA extraction kit (Cat. No. DP-302, TIANGEN, Beijing, China) following the manufacturer’s instructions for 16S rRNA gene profiling. The V3–V4 hypervariable regions of the 16S rRNA gene were amplified by PCR using specific primers (338 F: ACTCCTACGGGAGGCAGCA; 806 R: GGACTACHVGGGTWTCTAAT) and high-fidelity DNA polymerase. After quality control, amplicons were sequenced on an Illumina Novaseq6000 platform (San Diego, CA, USA) to generate high-resolution profiles of the 16S V3–V4 regions.2.5.

#### 2.4.1 Random Forest Machine Learning

Random Forest machine learning was performed as previously described [14], [32]. Briefly, microbial genera were scored based on their importance using a random forest model (R 4.4.1, randomForest 4.7–1.1 package) subjected to 10-fold cross-validation. The selected set of genera was used to calculate the probability of the target condition, and a receiver operating characteristic (ROC) curve was generated using the pROC 1.18.5 package. The model was tested on the test set, and prediction error was evaluated. The model was further validated on an external dataset to assess its accuracy and generalizability. Script for Machine Learning were provided in Supplementary Methods.

### 2.5 Optimal microbial biomarker selection and biletype-driven clustering

The predictive efficacy of individual bacterial genera for CCA was evaluated based on the area under the ROC curve (AUC). Genera were ranked in descending order of AUC values, with the minimum AUC among the top 30 genera being 0.67. AUC calculation referenced the equivalence principle of Wilcoxon-Mann-Whitney test, which assesses the probability of correct ranking between positive and negative samples. Subsequently, combinations of the top 1–30 genera were sequentially selected as feature inputs [14] for unsupervised hierarchical clustering analysis to identify the optimal number of genera [16]. Unsupervised hierarchical clustering and visualization were performed using the Hiplot platform (https://hiplot.com.cn/cloud-tool/drawing-tool/detail/106) with the Ward.D2 algorithm applied to Euclidean distances. The Ward.D2 method merges clusters by minimizing within-cluster variance. The optimal cutoff was determined based on dendrogram morphology and the correlation between sample classification and clinical phenotypes (BBD/CCA), resulting in two distinct categories: Biletype A and Biletype B.

To validate the clustering efficiency, the identified cutoff values were applied to subgroup Biletype samples. Since Biletype A lacks CCA cases, associations between Biletypes and pathological staging or prognosis were only assessed within Biletype B. Unsupervised hierarchical clustering was performed on Biletype B samples to determine whether the cutoff values could further stratify patients into subcategories with statistically significant associations to clinical phenotypes and prognosis.

To further evaluate the predictive utility of the cutoff values, unsupervised hierarchical clustering was applied to assess whether the selected microbial genera could be perfectly clustered into two categories. The correlations between genus classification, Biletypes, sub-Biletypes, and clinicopathological factors were also explored.

### 2.6 Fluorescence In Situ Hybridization (FISH)

Tissue Section (3.5 µm) were baked at 65°C for 2 hours, followed by deparaffinization in xylene through three washes, each lasting 10 minutes. The sections were then dehydrated through an ethanol gradient (100 %, 85 %, and 70 %). Bacterial lysis was ensured by treatment with Proteinase K (Cat. No. 9034, Takara, Japan) and lysozyme (Cat. No. 8120, Solarbio, China). A hybridization buffer (900 mM NaCl, 20 mM Tris–HCl [pH 8.0], 0.01 % sodium dodecyl sulfate, and 30 % formamide) was prepared, and the EUB 338 probe was diluted to a final concentration of 5 ng/µl. The probe was denatured and prehybridized before being applied to the sections in a humid chamber at 37°C for 24 hours. Post-hybridization washes were performed, and sections were air-dried and mounted with DAPI (Cat. P0131, Beyotime, China) for imaging. A confocal laser scanning microscope (Nikon, Tokyo, Japan) was used to capture images of EUB338-stained microorganisms in tissue sections, where positive staining indicates bacterial presence, as previously described [16], [33].

### 2.7 Statistical analysis

Statistical analyses were performed using R package and IBM SPSS Statistics 26.0. Quantitative data are expressed as mean ± standard deviation (SD). One-way analysis of variance (ANOVA) or independent Student’s *t*-test was used to compare the quantitative data. The Kaplan-Meier test was employed to identify risk factors. A two-sided P-values < 0.05 was considered statistically significant. Graphpad prism 8.0 was used for data visualization.

## 3 Results

### 3.1 Intratumoral microbiota

**Fig. 1:**
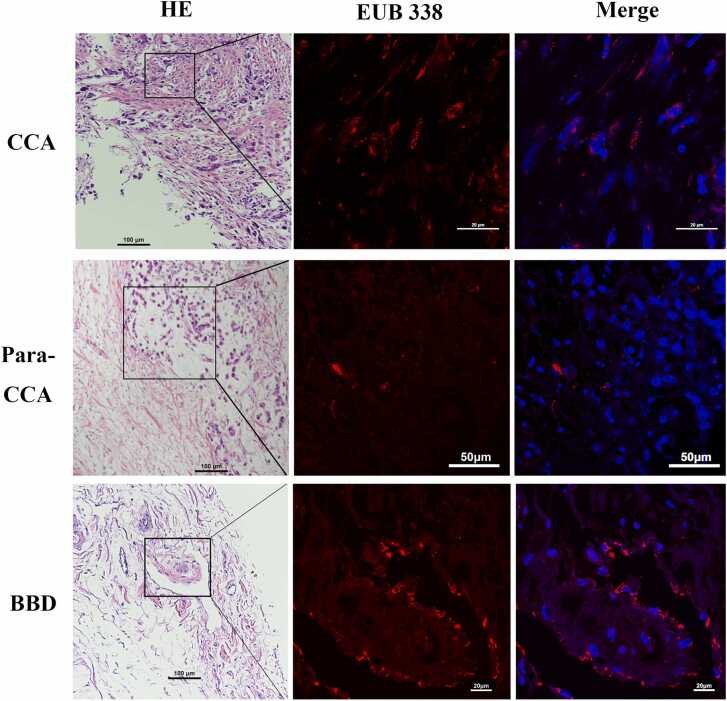
FISH images**.** EUB 338 probe labeled by red fluorescence was used to indicate bacterial presence. The left panel illustrates an H&E-stained image, the middle panle highlights bacterial presence with the EUB 338 probe in the same region, and the right integrates both into a composite image.

Bacterial presence was consistently observed in CCA, para-CCA, and BBD (Fig. 1) samples. Among five paired CCA and BBD tissue samples, bacteria were detected in 100 % of CCA tissues and 80 % of BBD tissues, confirming their presence in both tissue types. Bacteria were localized within cholangiocytes or in the pericellular microenvironment of the biliary tract tissue (Fig. 1). These findings suggest that bacteria play a role in the development of CCA and may be involved in the pathogenesis of the disease.

### 3.2 Cohort selection and characteristics

**Fig. 2:**
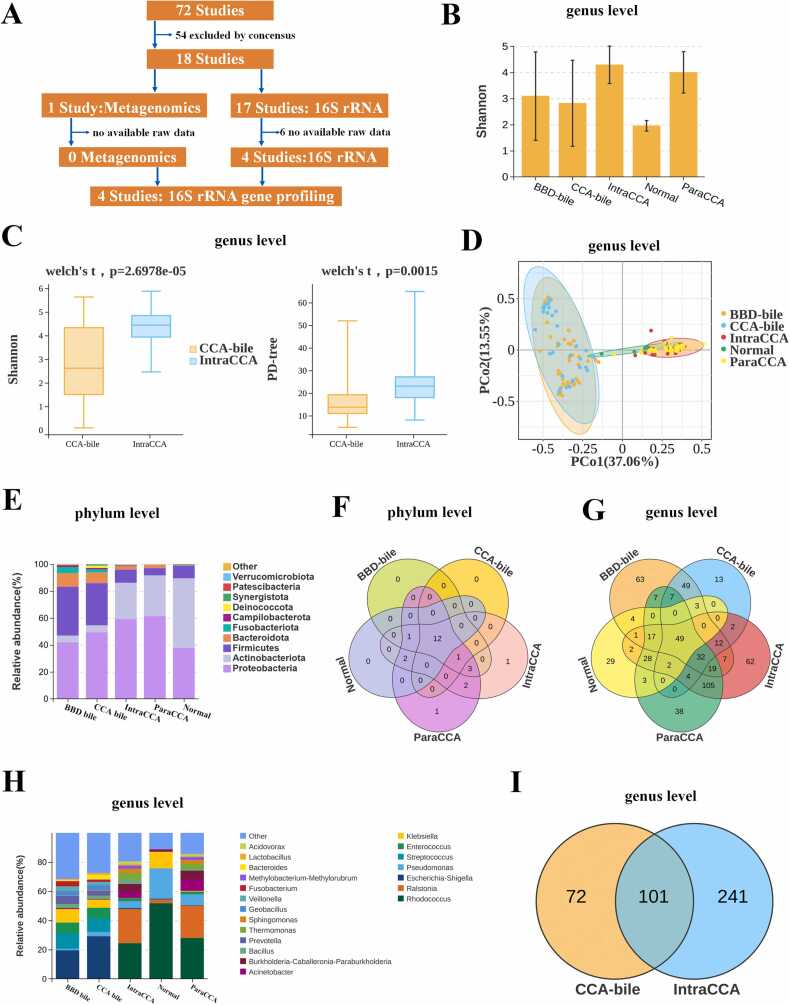
Differential microbiota composition among the five groups across Cohorts 1, 2, and 3. (A) Selection criteria and cohort processing methodology. (B) Shannon index at the genus level were analyzed among the five groups. (C) α-diversity at the genus level calculated by Shannon index and PD-tree index was markedly higher in IntraCCA compared to CCA bile. (D) β-diversity at the genus level was analyzed using PCoA among the five groups. (E) Stacked bar plots of phylum abundance among the five groups. (F, G) Venn analysis of phylum- and genus-level differences among five groups. (H) Stacked bar plots depicting genus-level community composition among five groups. (H) A total of 101 genera were shared between CCA bile and IntraCCA.

To investigate the origins and transformation processes of bacteria within biliary tissue, we annotated and analyzed raw data from Cohorts 1, 2, and 3, all of which utilized 16S rRNA gene profiling to differentiate CCA from BBD (Fig. 2a, Table 1). Cohort 1 included 4 CCA bile and 3 BBD bile samples, Cohort 2 comprised 28 CCA bile and 47 BBD bile samples, and Cohort 3 included 45 CCA tissues, 49 adjacent non-tumorous tissues, and 5 normal liver tissues (Table 2). These three cohorts represent populations from Japan, Iran, and China. After controlling for batch effects, the microbial community distribution in each cohort aligns with the results of the original studies (Supplementary Fig. 1a-c). The consistency in microbial community distribution across different cohorts suggests that the observed differences in microbiota are not due to batch effects but rather reflect true biological variations.

**Table 2:** Distribution of cases in five Cohorts.

	CCA bile	BBD bile	IntraCCA tissue	ParaCCA tissue	Normal
Cohort 1	4	3			
Cohort 2	28	47			
Cohort 3			45	49	5
Cohort 4	26	17			
Cohort 5	19	23			

CCA cholangiocarcinoma, BBD benign biliary disease, Normal normal liver tissue

### 3.3 α-diversity and β-diversity

Patients in Cohorts 1, 2 and 3 were categorized into five groups: CCA bile, BBD bile, intratumor tissue (IntraCCA), paratumor tissue (ParaCCA), and normal hepatic tissue (Normal). At the genus level, the Shannon index revealed significantly lower α-diversity in the Normal group compared to the other four groups (Fig. 2b, Supplementary Fig. 2a-d). The IntraCCA group exhibited higher α-diversity at the genus level than the CCA bile group (Fig. 2c). Principal Coordinate Analysis (PCoA) demonstrated significant differences in microbial community composition at the genus level between CCA bile and IntraCCA tissue (Fig. 2d**)**. Moreover, the distribution of the microbial community of genus level in the Normal group significantly differed from the other groups (Fig. 2d**)**, indicating a microbiota shift from healthy to BBD and CCA states.

### 3.4 Community Composition of Bile and Intra-tumoral Microbiota

Significant differences were observed at the phylum level between CCA bile and IntraCCA tissue (Fig. 2e). The six dominant phyla in CCA bile were *Proteobacteria*, *Firmicutes*, *Bacteroidota*, *Actinobacteriota*, *Fusobacteriota*, and *Deinococcota*, while IntraCCA tissue was dominated by *Proteobacteria, Actinobacteriota*, *Firmicutes*, *Bacteroidota*, *Patescibacteria*, and *Verrucomicrobiota*. Notably, 12 phyla, including *Proteobacteria*, *Firmicutes*, *Bacteroidota*, and *Actinobacteriota*, were consistent across all five groups (Fig. 2f), aligning with previous findings [34], suggesting no major disruption in biliary and intratumoral microbiota.

**Fig. 3:**
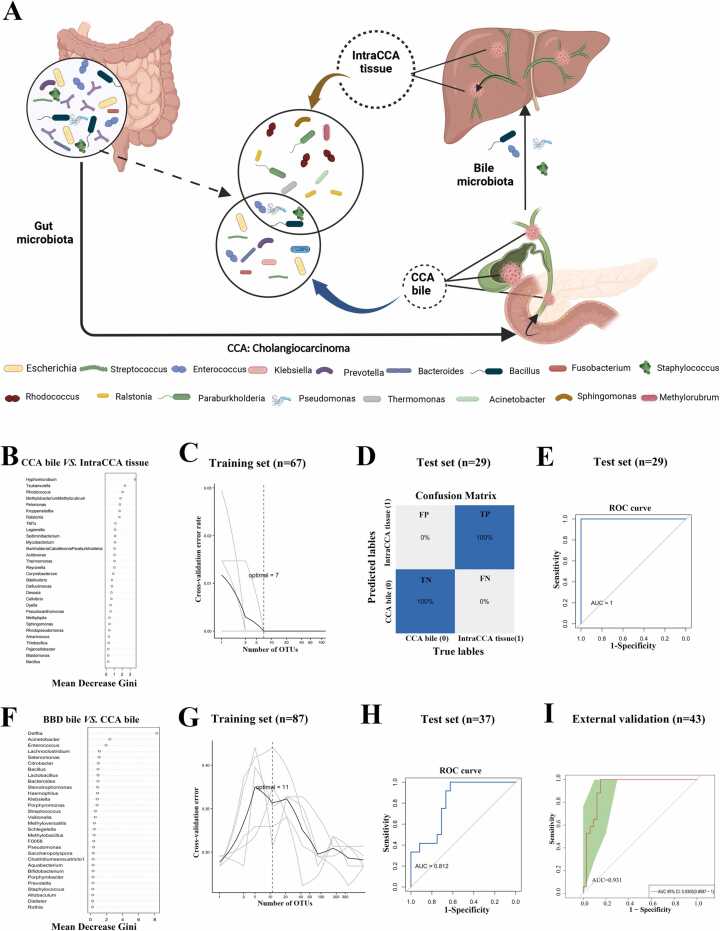
Schematic diagram of biliary microbiota migration and Random Forest Machine Learning. **(A)** Gut microbiota migrated to the bile and some bile microbiota subsequently migrated into CCA tissue. Enterococcus, Pseudomonas, Bacillus and Staphylococcus were representatives. The names of these genera were listed at the base of the figure. (B) Ranking the importance of CCA tissue-associated microbiota for differentiating from CCA bile. (C) Five repetitions of 10-fold cross-validation in the training set led to the optimal selection of 7 biomarker genera for differentiating intraCCA tissue from CCA bile.(D) Confusion matrix in the test set showed perfect efficiency for differentiating intraCCA tissue from CCA bile, with 100 % true positive (TP) rate and 100 % ture negative (TN) rate. (E) ROC curve in the test set showed perfect result for differentiating intraCCA tissue from CCA bile, with AUC of 1.0. The grey line represents the ROC curve for a random guess. Higher AUC value indicates better performance of the model. (F) The 30 genera that contributed most significantly to the classifier for predicting CCA. (G) Five repeats of 10-fold cross-validation on the training set resulted in the optimal selection of 11 biomarker genera for predicting CCA. (H) ROC curve in the test set for predicting CCA with an AUC of 0.812. The grey line represented the ROC curve for a random guess. Higher AUC value indicated better performance of the model. (I) ROC curve of external validation for predicting CCA achieved an AUC of 0.931. The grey line represented the ROC curve for a random guess. The area under the red curve represented the AUC value. The green shaded region indicated the 95 % confidence interval for the AUC value. Higher AUC value indicated better performance of the model.TP, true positive; TN, true negative; FP, false positive; FN, false negative. AUC, Area Under the Curve.

At the genus level, 49 genera were shared by all five groups (Fig. 2g). However, significant discrepancies were observed between CCA bile and IntraCCA tissue (Fig. 2h and Fig. S2e). The top 11 predominant genera of CCA bile included *Escherichia*, *Streptococcus*, *Enterococcus*, *Klebsiella*, *Prevotella*, *Bacteroides*, *Geobacillus*, *Pseudomonas*, *Veillonella*, *Bacillus*, and *Fusobacterium*. In contrast, the top eleven predominant genera in IntraCCA tissue included *Rhodococcus*, *Ralstonia*, *Paraburkholderia*, *Pseudomonas*, *Thermomonas*, *Acinetobacter*, *Bacillus*, *Sphingomonas*, *Methylorubrum*, *Enterococcus*, and *Acidovorax*. Among the top 11 genera, *Enterococcus*, *Pseudomonas*, and *Bacillus* were shared between CCA bile and IntraCCA tissue. Moreover, across all genera，a total of 101 genera were shared between CCA bile and IntraCCA tissue. (Fig. 2i), suggesting microbiota migration from bile to tumor tissue. The CCA bile microbiome resembled the gut microbiome in colorectal cancer, with abundant *Escherichia*, *Bacteroides*, *Enterococcus*, and *Fusobacterium*, indicating a gut origin [9], [14]. The intratumor microbiome, dominated by *Paraburkholderia* and *Acinetobacter*, resembled that of primary liver cancer [32]. These findings suggest that a subset of gut microbiota migrates to the bile, and a portion of bile microbiota subsequently integrates into tumor tissue, forming a distinct microbial community (Fig. 3a).

### 3.5 Distinguishing intratumoral microbiota from bile using machine learning

A total of 51 CCA bile samples and 45 IntraCCA tissue samples from Cohorts 1, 2, 3 and 4 were randomly allocated into training and test sets in a 7:3 ratio. The top 30 genera contributing most to the classifier in the training set are shown in Fig. 3b. Five repeats of 10-fold cross-validation (Fig. 3c) in the training set identified 7 optimal biomarker genera. The training set confusion matrix revealed a high number of true negatives (TN) and true positives (TP) compared to false positives (FP) and false negatives (FN) (Supplementary Fig. 3a)**.** A similar trend was observed in the test set (Fig. 3d). The ROC curve showed an AUC of 1.0 for both the training (Supplementary Fig. 3b) and test sets (Fig. 3e), indicating that the combination of these 7 genera effectively distinguishes intratumoral microbiota from biliary microbiota in CCA. Due to the low incidence of CCA and limited availability of tissue sequencing data, external validation was not feasible.

### 3.6 Predicting CCA by machine learning

Similarly, 51 CCA bile samples and 73 BBD bile samples from Cohorts 1, 2, and 5 were randomly assigned into training and test sets in a 7:3 ratio. The top 30 genera contributing most to the classifier are shown in Fig. 3f. Five repetitions of 10-fold cross-validation (Fig. 3g) selected 11 biomarker genera. The AUC value for the training set was 1.0 (Supplementary Fig. 3c), while the test set achieved an AUC of 0.812 (Fig. 3h). Cohort 4, comprising 26 CCA bile and 17 BBD bile samples from the U.S., was used for external validation. The ROC curve for external validation achieved an AUC of 0.931 **(**Fig. 3i**)**, demonstrating strong predictive performance. These results suggest that the machine learning model can effectively distinguish CCA bile from BBD bile, and that the selected biomarker genera have good predictive value.

### 3.7 Biletypes based on bile microbiome

**Fig. 4:**
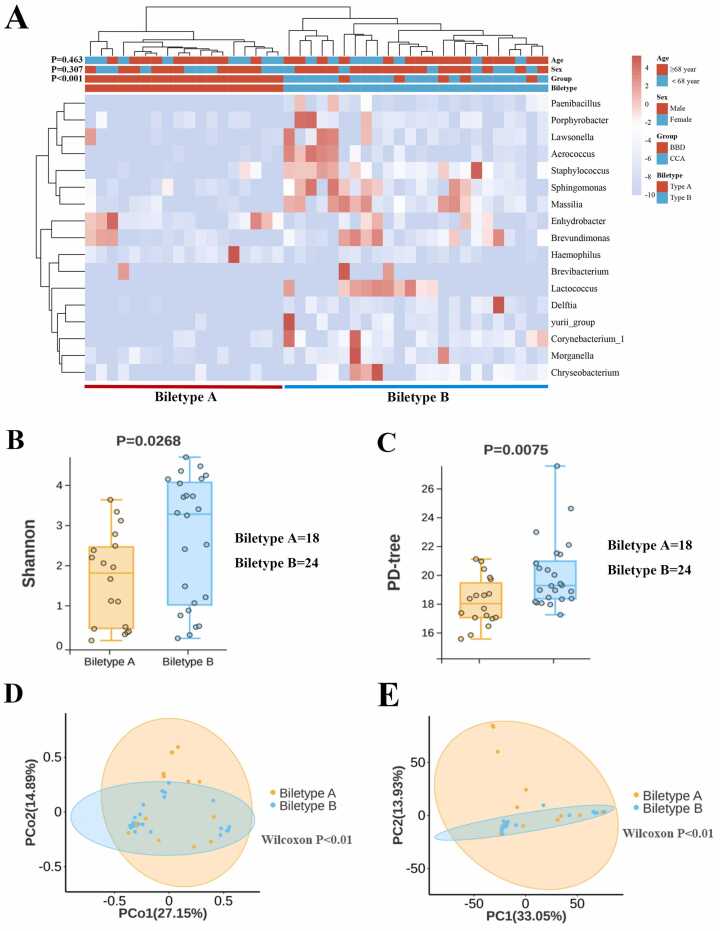
Biletypes and community composition analysis in Cohort 5. (A) Combinations of the top 1–30 genera were selected as feature inputs for unsupervised hierarchical clustering analysis to identify the optimal number of genera. The combination of the top 17 genera achieved the optimal hierarchical clustering effect by clustering the 42 cases in Cohort 5 into Biletype A and Biletype B. Biletype B was significantly associated with CCA. (B, C) Shannon index and PD tree index were analyzed between Biletype A and Biletype B. (D, E) β-diversity was analyzed using PCoA and PCA between Biletype A and Biletype B.

Establishing Biletypes maybe a novel way to predict CCA. To improve prediction accuracy for CCA, we focused on Cohort 5, which included 19 CCA and 23 BBD bile samples with complete clinicopathological and prognostic data from our institute. Baseline characteristics in Cohort 5 were well-balanced (Supplementary Table 1). The combination of the top 17 genera based on AUC values achieved optimal hierarchical clustering, dividing the 42 cases into Biletype A and Biletype B (Fig. 4a). Age and gender distributions were similar between the two Biletypes (P > 0.05). The proportion of CCA in Biletype B (79.2 %, 19/24) was significantly higher than that in Biletype A (0 %, 0/18) (P < 0.001) (Fig. 4a). Of the 17 genera, only *Enhydrobacter* and *Haemophilus* were enriched in Biletype A, while the other 15 genera, including *Sphingomonas*, *Staphylococcus*, and *Massilia*, were significantly enriched in Biletype B (Fig. 4a). Cross-validation with different subsets of Cohort 5 confirmed the stability of this classification (Supplementary Fig. 4a-b). Shannon and PD-tree indices were significantly higher in Biletype B (P < 0.005, Fig. 4b-c). PCA and PCoA analyses also showed significant differences between the two Biletypes (P < 0.01, Fig. 4d-e).

**Fig. 5:**
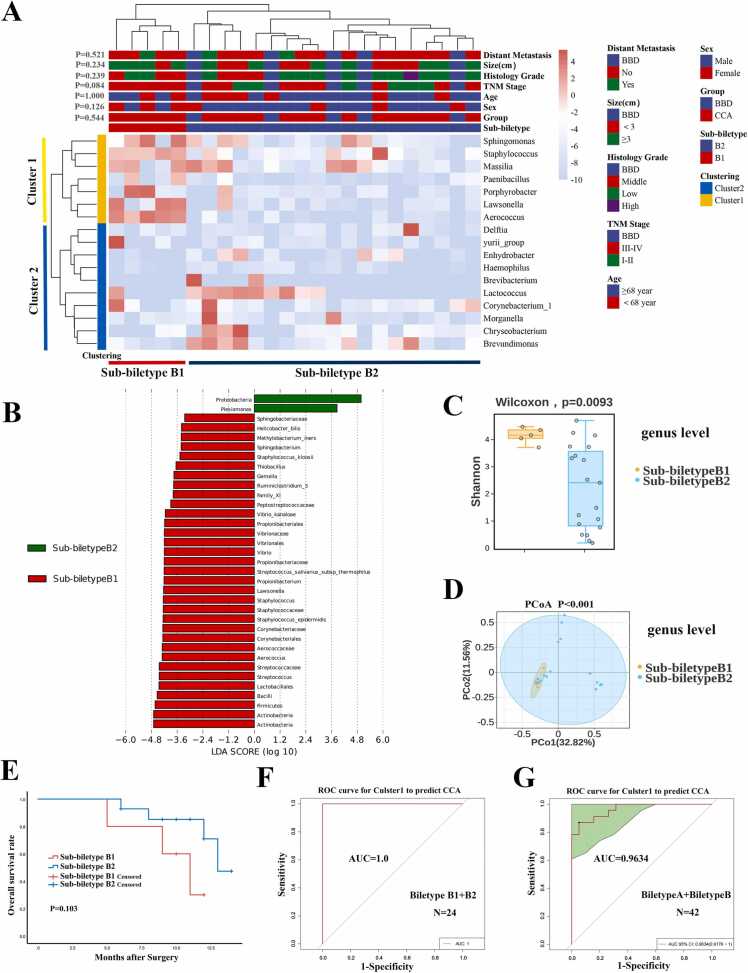
Sub-Biletypes and their correlation with clinicopathological factors, prognosis, and community composition in Cohort 5. (A) The 24 cases in Biletype B were sub-clustered into Sub-Biletype B1 and Sub-Biletype B2. Bacterial Cluster 1 including Sphingomonas, Staphylococcus, Massilia, Paenibacillus, Porphyrobacter, Lawsonella, and Aerococcus were significantly enriched in Sub-Biletype B1. Sub-Biletype B1 tended to be associated with advanced CCA (TNM III-IV). (B) Indicator species for CCA identified through LEfSe bar analysis. (C) The Shannon indice for Sub-Biletypes at the genus level. (D) β-diversity at the genus level was analyzed using PCoA and PCA between Sub-Biletypes. (E) Kaplan-Meier analysis of Sub-Biletypes. (F, G) ROC curve using Cluster 1 for predicting CCA were trained in 24 cases with Biletype B and validated in all 42 cases from Cohort 5. The grey line represents the ROC curve for a random guess. Higher AUC value indicates better performance of the model. The green area represents the 95 % CI of AUC value.

To further validate the clustering efficiency, we stratified Biletype B into Sub-Biletype B1 and Sub-Biletype B2 using the aforementioned 17 genera (Fig. 5a). All Sub-Biletype B1 samples (5/5) were CCA, compared to 14/19 in Sub-Biletype B2. Notably, 100 % of CCA cases in Sub-Biletype B1 were TNM stage III-IV, compared to 42.1 % (8/19) in Sub-Biletype B2 (P = 0.084), indicating higher malignant potential in Sub-Biletype B1 (Fig. 5a). No significant associations were found between subtypes in gender, age, histological grade, or distant metastasis (P > 0.05, Fig. 5a, Supplementary Table 2). Bacterial Cluster 1, including *Sphingomonas*, *Staphylococcus*, *Massilia*, *Paenibacillus*, *Porphyrobacter*, *Lawsonella*, and *Aerococcus*, was significantly enriched in Sub-Biletype B1 (Fig. 5a). LEfSe analysis identified *Sphingomonas*, *Staphylococcus*, *Lawsonella* and *Aerococcus* as characteristic of Sub-biletype B1 (Fig. 5b). At the genus level, Shannon and Simpson indexes were significantly higher in Sub-Biletype B1 (P < 0.001, Fig. 5c). PCoA analysis revealed significant β-diversity differences between the two subtypes (P < 0.01, Fig. 5d). Kaplan-Meier analysis suggested that Sub-Biletype B1 was potentially associated with poorer prognosis (Fig. 5e).

The ROC curve demonstrated that bacterial Cluster 1 predicted CCA with 100 % accuracy in Biletype B (Figs. 5f) and 96.3 % accuracy across all 42 patients including Biletype A and Biletype B. (Fig. 5g), highlighting its strong predictive power.

### 3.8 Staphylococcus serves as an optimal biomarker for distinguishing CCA from BBD

**Fig. 6:**
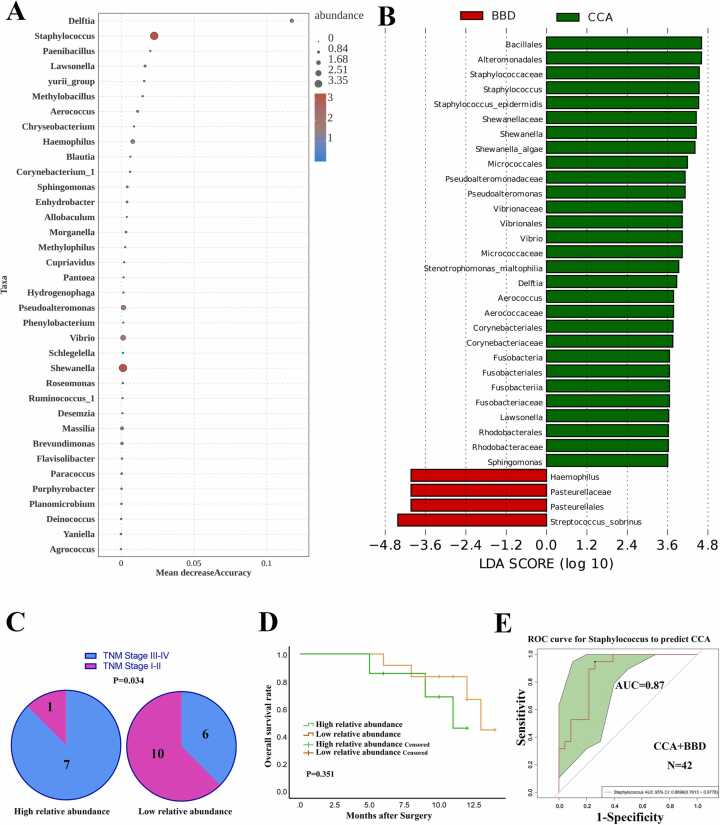
Identification of Staphylococcus as a unique biomarker for distinguishing CCA from BBD. (A) Random Forest analysis were conducted across all 42 cases in Cohort 5. (B) The LEfSe analysis were performed for all 42 cases in Cohort 5. Most significant OTUs with LDA > 3.6 were included in this analysis. (C) The correlation between the relative abundance of Staphylococcus and TNM stage was analyzed. (D) Kaplan-Meier analysis for overall survival based on varying abundances of *Staphylococcus* in Cohort 5. (E) The ROC curve demonstrated the strong predictive ability of *Staphylococcus* for CCA (AUC = 0.87). The grey line represents the ROC curve for a random guess. Higher AUC value indicates better performance of the model. The green area represents the 95 % CI of AUC value.

To pinpoint the most significant genus for predicting CCA, we performed RF (Fig. 6a) and LefSe analyses (LDA > 3.6, Fig. 6b) across all 42 cases categorized as CCA or BBD in Cohort 5. Intersecting RF and LefSe results with the seven Cluster 1 genera consistently identified *Staphylococcus* as the top genus, marking it as a key CCA indicator. Among patients with higher Staphylococcus abundance, 87.5 % (7/8) had advanced TNM stages, compared to 37.5 % (6/16) with lower abundance (P = 0.034) (Fig. 6c). Patients with higher *Staphylococcus* abundance tended to have poorer prognosis (Fig. 6d). The ROC curve confirmed *Staphylococcus* as an optimal biomarker for distinguishing CCA from BBD (Fig. 6e). Previous studies have demonstrated that Staphylococcus promotes tumor progression through immune evasion, neutrophil extracellular trap (NET) formation [35], [36], and degradation of tumor suppressors and microRNAs [37], [38]. Future research will experimentally validate its role in cholangiocarcinoma.

## 4 Discussion

Historically, the healthy biliary system was considered sterile. Previous researches have primarily focused on the gut microbiome's role in primary liver cancer [39], [40]. However, recent studies have revealed the presence of microbial ecosystems in the biliary tract, even in individuals without hepatobiliary disorders [41]. Notable examples of such pathogens include Salmonella spp. and Listeria monocytogenes [42], [43]. The colonization and disequilibrium of microbial communities in the biliary tract not only modulates the local inflammation but may also contribute to the pathogenesis of biliary tumors [44]. In this study, we observed significantly higher microbial alpha diversity in CCA bile and tissue compared to normal liver tissue, underscoring the biliary tract as a critical reservoir for bacteria in tumor development.

Identifying microbial biomarkers across cross-cohort datasets is challenging due to batch effects, which can obscure true signals and generate false positives [24], [45]. Methods such as Conditional Quantile Regression (ConQuR) [24], ComBat [46], and MMUPHin [47] have been developed to mitigate these effects, but limitations remain. Despite all sequencing being conducted on Illumina platforms, variations in region, bile sampling, bacterial DNA extraction, and PCR amplification could affect microbial composition. To address this, In this study, bile samples in this study were collected during surgery and immediately snap-frozen in liquid nitrogen to preserve integrity. Primers targeting the V3-V4 region were utilized, and normalization was performed based on the minimum abundance value. These measures were carefully designed to minimize batch effects. Internal and external validation, following the principles of the xMarkerFinde [25] further minimized batch effects, yielding promising results in distinguishing IntraCCA tissue microbiota from CCA bile microbiota, as well as distinguishing CCA bile microbiota from BBD bile microbiota.

The Random Forest (RF) model achieved 100 % accuracy and specificity in predicting intra-CCA tissue from CCA bile. Although extensive internal validation was performed, external validation was not feasible due to limited external cohorts. Future efforts will focus on systematically obtaining raw data from newly established researches to construct external validation. The decline in accuracy and specificity when predicting CCA bile from BBD bile suggests the RF model is objective and not overfitted. Similar predictive outcomes have been reported in colorectal cancer using analogous methodologies [48], further validating our approach.

The combination of seven genera in Cluster 1 (*Sphingomonas*, *Staphylococcus*, *Massilia*, *Paenibacillus*, *Porphyrobacter*, *Lawsonella*, and *Aerococcus*) successfully predicted CCA diagnosis, staging, and prognosis. Genera such as *Sphingomonas*, *Staphylococcus*, and *Massilia* have been linked to tumor progression [49]. Using clustering analysis, random forest, and LEfSe methodologies, we identified Staphylococcus as a key indicator species and established its correlation with clinicopathological features and prognosis in CCA. ROC curve analysis demonstrated that bile *Staphylococcus* alone could effectively predict CCA. Bile samples, obtained through invasive methods like gallbladder puncture, PTCD, or endoscopic Retrograde Cholangiopancreatography, can be analyzed using 16S rRNA to predict CCA with Cluster 1 or *Staphylococcus*. Although invasive, these procedures are commonly performed in patients with suspected biliary diseases, making bile microbiota analysis a useful diagnostic tool [50], [51]. Advances in minimally invasive techniques further support the feasibility of bile microbiota analysis.

However, invasive sampling requires informed consent and complication management. Given the similarity between bile and gut microbiota, stool microbiota analysis offers a non-invasive alternative for predicting CCA. Stool sample collection is non-invasive and can be easily performed at home or in clinical settings, making it highly accessible. The stability of microbial DNA in stool samples allows for flexible storage and transportation conditions, facilitating widespread use [52], [53]. The decreasing costs of high-throughput sequencing and standardized protocols for DNA extraction and bioinformatics analysis have made microbiota analysis more accessible [54], [55]. Early and accurate diagnosis of CCA through microbiota analysis could reduce the need for invasive procedures and improve patient outcomes, offering significant healthcare savings.

*Staphylococcus*, commonly found on the human body surface and in the gut, may disrupt the intestinal mucosal barrier, enter the bloodstream, or release carcinogenic factors affecting distant organs. It may also reach the hepatobiliary system via the enterohepatic circulation or biliary tract [56]. *Staphylococcus aureus* has been linked to tumor formation through immune evasion [49], [57], while specific clones of *Staphylococcus lugdunensis* are associated with colorectal cancer [13]. *Staphylococcus epidermidis* has been genetically engineered for cancer prevention or treatment [58]. Mechanistically, *Staphylococcus aureus* may exert pro-tumor effects through staphylococcal nuclease (SNase), a genomic marker for *Staphylococcus aureus*. Its human homolog, SND1 (staphylococcal nuclease and Tudor domain-containing 1), is an oncoprotein that regulates transcription, mRNA splicing, RNAi function, and mRNA stability [37]. *Staphylococcus*-derived SND1 accelerates tumor progression by forming an RNA-induced silencing complex, targeting tumor suppressors like PTEN and miRNAs [37], [38]. Another potential mechanism involves Staphylococcus and its virulence factors, such as nuclease, Eap, and FnBPB, promoting tumor metastasis by inducing NETs [35], [36]. During Staphylococcus infection, non-lytic inflammatory cell death of neutrophils leads to the rapid release of chromatin and enzymes, forming extracellular NETs [59]. While NETs can trap circulating tumor cells, their enzymatic components have limited cytotoxic effects on tumor cells, potentially promoting metastasis. Studies show that NET-derived elastase and matrix metalloproteinase-9 can reactivate dormant micrometastases in mice [60], and NET DNA can interact with CCDC25 on tumor cells to facilitate metastasis [61]. We hypothesize that Staphylococcus interactions with the tumor immune microenvironment may influence CCA progression and that engineering Staphylococcus as a therapeutic strain could offer a novel treatment strategy. Future studies will investigate these pathways in biliary tract tumors.

Therapeutically, this study identifies indicator species that could guide the integration of specific antibiotics into CCA treatments. For example, eliminating Fusobacterium with metronidazole has been shown to enhance immunotherapy efficacy [9]. Anti-*Staphylococcus* regimens may activate immune cells to kill tumor cells[49], potentially boosting chemotherapy efficacy. Combining anti-Staphylococcus regimens with oncological therapies offers a promising strategy to improve treatment outcomes. Although Staphylococcus is typically susceptible to penicillin, β-lactamase production necessitates the use of β-lactamase inhibitor combinations.

## 5 Conclusions

Our multi-cohort approach enhances the generalizability of findings and provides a comprehensive comparison across distinct patient populations. Using machine learning, we identified specific microbiota signatures associated with cholangiocarcinoma, advancing diagnostic and prognostic accuracy. Bacterial Cluster 1, including *Sphingomonas*, *Staphylococcus*, *Massilia*, *Paenibacillus*, *Porphyrobacter*, *Lawsonella*, and *Aerococcus*, successfully predicted CCA from BBD. The identification of Staphylococcus as a diagnostic and prognostic biomarker for CCA represents a significant breakthrough with implications for patient management and therapeutic strategies. The novel concept of combining antibiotics with anticancer drugs to enhance therapeutic outcomes could transform treatment paradigms for CCA patients.

Additional experiments were not feasible in this study. Future research in larger cohorts is needed to confirm these findings and explore the role of bile microbiota in CCA development. Clinical trials are also necessary to test the efficacy of microbiota-targeted therapies in CCA patients. These findings open new avenues for understanding CCA etiology and suggest that manipulating bile microbiota could be a potential strategy for CCA prevention or treatment. Further research is needed to explore the causal relationships and mechanisms underlying these associations.

## Ethics approval

Our center's clinical cohort data and publically accessible data were integrated in this study. All subjects gave their informed consent, and the initial 16S rRNA sequencing investigations used in the RF machine learning study were approved by the ralevant ethical committees. The Ethics Committees of Jiangnan University Medical Center (Approval No. 2023-Y-33) and Nanjing Medical University (Approval No. 2019–784) have approved research involving human specimens in cohort 4. Written informed consent was given by each participant in the study from our center.

## Funding

This work was supported by Qinghai Province Key Research and Development and Transformation Plan Specific fund of Science and Technology Assistance to Qinghai (No. 2022-QY-216), the Wuxi "Double Hundred" Young and Middle-aged Top Medical and Health Talents Project (HB2023030), and the Science Foundation of Health Commission of Wuxi (No.Q202126).

## CRediT authorship contribution statement

**Yang Mengmeng:** Writing – review & editing, Software, Resources, Methodology, Formal analysis, Data curation. **Wen Sijia:** Writing – review & editing, Software, Resources, Investigation. **Wang Lei:** Writing – review & editing, Writing – original draft, Software, Methodology, Investigation, Funding acquisition, Formal analysis, Data curation, Conceptualization. **Chen Daozhen:** Writing – review & editing, Supervision, Resources, Methodology, Funding acquisition, Conceptualization. **Chen Yu:** Writing – review & editing, Writing – original draft, Supervision, Methodology, Conceptualization. **Xia Jiazeng:** Writing – review & editing, Supervision, Resources, Conceptualization. **Xu Hanjie:** Writing – review & editing, Software, Methodology, Formal analysis, Data curation. **Chen Jiale:** Writing – review & editing, Software, Resources, Methodology, Data curation. **Wu Fan:** Writing – review & editing, Software, Resources, Methodology, Investigation, Formal analysis. **Zhao Hui:** Writing – review & editing, Writing – original draft, Resources, Methodology, Investigation, Funding acquisition, Formal analysis, Conceptualization. **Gong Wanwan:** Writing – review & editing, Visualization, Resources, Investigation.

## Declaration of Interest

None.

## Footnotes

Appendix A Supplementary data associated with this article can be found in the online version at doi:10.1016/j.csbj.2025.03.030.
