# Supplementary material for: Bile-Liver phenotype: Exploring the microbiota landscape in bile and intratumor of cholangiocarcinoma
Source: Comput Struct Biotechnol J. 2025 Mar 18;27:1173–86. doi: 10.1016/j.csbj.2025.03.030 (PMC11981758; doi:10.1016/j.csbj.2025.03.030)
Supplement: Supplementary file 1 — Supplementary material [file mmc1.docx]

**
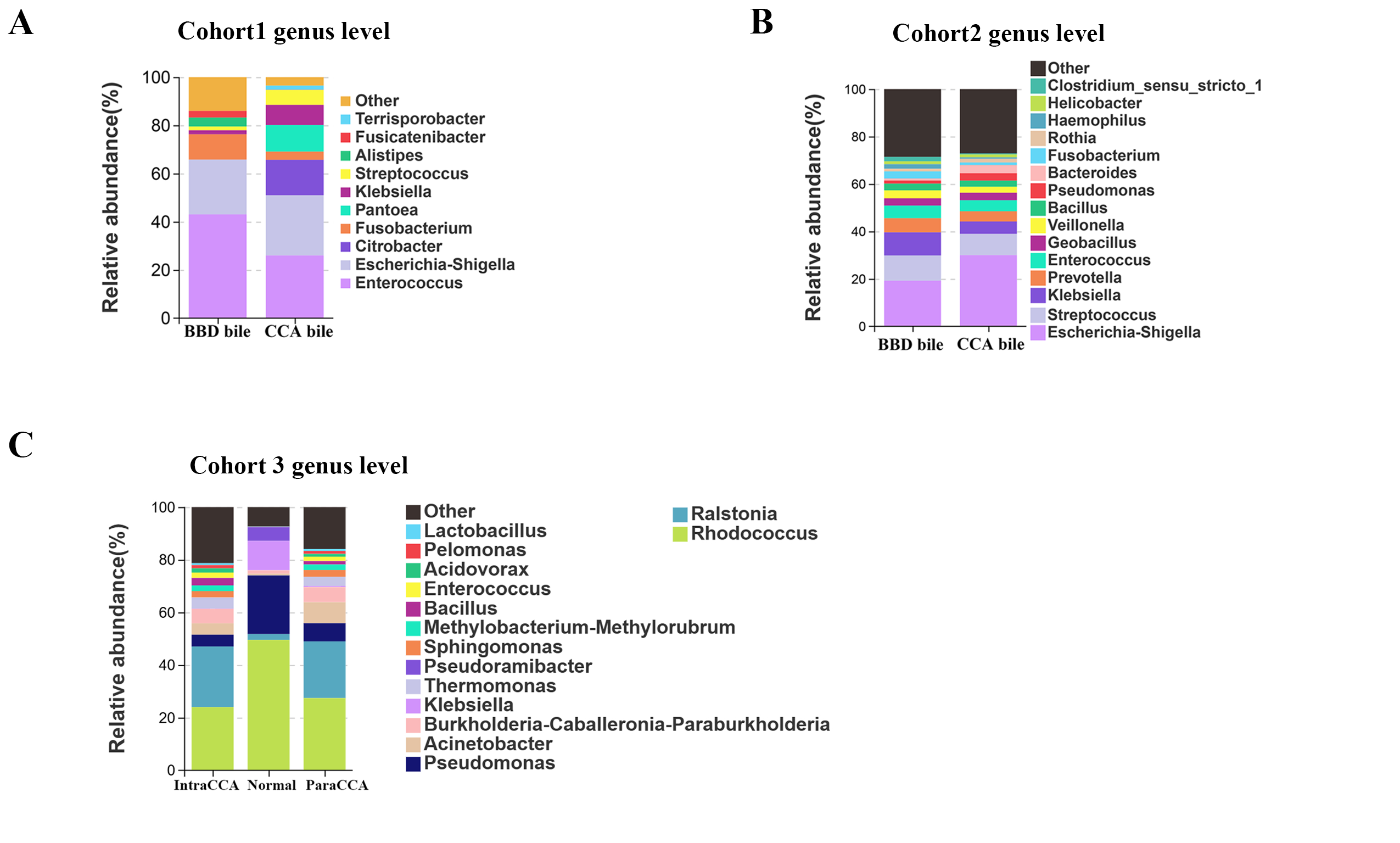
**

**Fig. S1** Uniform processing of 16S rRNA gene profiling. The taxonomic compositions at the genus level for Cohort 1 (A), Cohort 2 (B) and Cohort 3 (C) are shown, closely matching the original studies’ findings.

**Supplementary Fig. 2.** alpha-diversity and community composition across five groups in Cohorts 1, 2, and 3. **a-d** α-diversity at the genus level was markedly higher in BBD bile, CCA bile, IntraCCA tissue and ParaCCA tissue compared to the Normal group. **e** Stacked bar plots depicting genus-level community composition.

**Supplementary Fig. 3** Confusion matrix and ROC curve. **a** Confusion matrix in the training set for differentiating intraCCA tissue from CCA bile. **b** The ROC curve showed an AUC value of 1.0 for differentiating intraCCA tissue in the training set. **c** The ROC curve showed an AUC value of 1.0 for predicting CCA from BBD in the training set. The grey line represents the ROC curve for a random guess. The blue line represents the calculated ROC curve and higher AUC value indicates better performance of the model.


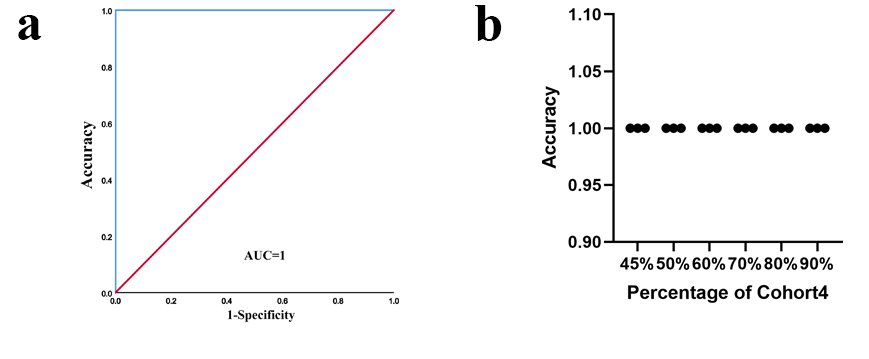


**Supplementary Fig. 4** Cross-validation with cases from different subsets of Cohort 4. **a** ROC curve of cross-validation with 45% cases of Cohort 4. The red line represents the ROC curve for a random guess. The blue line represents the calculated ROC curve and higher AUC value indicates better performance of the model. **b** AUC values of cross-validation from different subsets of Cohort 4
