# Supplementary material for: Bile-Liver phenotype: Exploring the microbiota landscape in bile and intratumor of cholangiocarcinoma
Source: Comput Struct Biotechnol J. 2025 Mar 18;27:1173–86. doi: 10.1016/j.csbj.2025.03.030 (PMC11981758; doi:10.1016/j.csbj.2025.03.030)
Supplement: Supplementary file 2 — Supplementary material [file mmc2.docx]

**SUPPLEMENTARY METHODS**

**Search query**

((Gallbladder cancer[Title/Abstract] OR biliary tract cancer[Title/Abstract] OR hepatobiliary cancer[Title/Abstract] OR hepatopancreatobiliary cancer[Title/Abstract] OR biliary tract carcinoma[Title/Abstract] OR bile duct cancer[Title/Abstract] OR bile duct carcinoma[Title/Abstract] OR gall bladder cancer[Title/Abstract] OR gallbladder carcinoma[Title/Abstract] OR gall bladder carcinoma[Title/Abstract] OR extrahepatic cholangiocarcinoma[Title/Abstract] OR intrahepatic cholangiocarcinoma[Title/Abstract] OR cholangiocarcinoma[Title/Abstract]) AND (Microbio[Title/Abstract] OR microbial[Title/Abstract] OR microbiome[Title/Abstract] OR microbiota[Title/Abstract] OR dysbacteriosis[Title/Abstract] OR microbiota disbiosis[Title/Abstract])) NOT (review)

**The Role and Mechanism of the Random Forest Model and** **Script for Machine Learning**

The random forest model is a decision-tree-based machine learning algorithm that excels in handling complex variable interactions through a nonlinear risk function without the need for prior assumptions [1]. Furthermore, RF is particularly advantageous for modeling outcomes based on genomic data, effectively leveraging numerous features, even when explicit data are scarce [2].

###初始化设置

# 清除环境变量

rm(list=ls())

# 加载必要的库

library(dplyr)

library(data.table)

library(randomForest)

library(caret)

library(pROC)

library(ggplot2)

library(ggpubr)

library(ggprism)

library(tidyverse)

###数据加载和预处理

set.seed(123)

# 加载数据集

dat1 <- read.table("Genus.csv", header=TRUE, sep=',', row.names = 1)

conf <- read.table("sample.csv", header=TRUE, sep=',', row.names = 1)

# 计算相对丰度（这里使用对数标准化）

dat1 <- log10(dat1 + 1) / log10(max(dat1))

# 提取共同的样本名

gid <- intersect(colnames(dat1), rownames(conf))

# 筛选共同样本的数据

dat1 <- dat1[, pmatch(gid, colnames(dat1))]

conf <- conf[pmatch(gid, rownames(conf)),]

# 筛选特定组别的数据

dat2 <- dat1[, conf$group %in% c("DZ", "LN")]

conf2 <- conf[conf$group %in% c("DZ", "LN"),]

# 转换组别标签

conf2$group <- as.factor(as.character(conf2$group))

outcome <- sub("DZ", "0", sub("LN", "1", as.character(conf2$group)))

outcome <- as.factor(outcome)

# 准备数据

dat <- dat2

X <- as.data.frame(t(dat))

X$outcome <- outcome

###模型训练

# 划分训练集和测试集

ind <- sample(2, nrow(X), replace=TRUE, prob=c(0.7, 0.3))

ind.train <- X[ind == 1,]

ind.test <- X[ind == 2,]

#如果 outcome 不是因子类型，即使它实际上代表分类数据，你也需要将其转换为因子类型

ind.train$outcome <- as.factor(ind.train$outcome)

# 寻找最优的mtry值

set.seed(100)

mtry_range <- 1:(ncol(ind.train)-1)

errRate <- sapply(mtry_range, function(i) {

mtry_fit <- randomForest(outcome ~ ., data=ind.train, mtry=i)

mean(mtry_fit$err.rate[,1])

})

m_opt <- which.min(errRate)

# 之后选择ntree值，ntree指定随机森林所包含的决策树数目，默认为500,

set.seed(100)

ntree_fit <- randomForest(outcome ~ ., data = ind.train, mtry = m_opt, ntree = 10000)

plot(ntree_fit) # ntree到100以后就基本不变

# 根据以上结果，默认情况下的mtry效果更好，所以以mtry=18,ntree=600为参数构建随机森林模型。

rf.train <- randomForest(outcome ~ ., data = ind.train, mtry = m_opt,

importance = TRUE,proximity=TRUE,ntree = 100)

# 绘制模型误差图

plot(rf.train)

print(rf.train)

round(importance(rf.train), 2)

varImpPlot(rf.train, main="Variable Importance")

### 交叉验证

set.seed(123)

#' rfcv是随机森林交叉验证函数：Random Forest Cross Validation

result <- rfcv(X[,-ncol(X)],X$outcome,cv.fold = 10)

result$error.cv #' 查看错误率表，21时错误率最低，为最佳模型

#' 绘制验证结果

with(result,plot(n.var,error.cv,log="x",type = "o",lwd=2))

# 使用replicate进行多次交叉验证，可选

result <- replicate(5, rfcv(ind.train[,-ncol(ind.train)],ind.train$outcome,cv.fold = 10), simplify=FALSE)

error.cv <- sapply(result, "[[", "error.cv")

error.cv <- cbind(rowMeans(error.cv),error.cv)

n.var = rownames(error.cv) %>% as.numeric()

error.cv = error.cv[,2:6]

colnames(error.cv) = paste('err',1:5,sep='.')

err.mean = apply(error.cv,1,mean)

allerr = data.frame(num=n.var,err.mean=err.mean,error.cv)

# number of features selected

optimal = 37

# 表格输出

write.table(allerr, file = "family_rfcv.txt", sep = "\t", quote = F, row.names = T, col.names = T)

# the pre-setted parameters used for ploting afterwards

main_theme = theme(panel.background=element_blank(),

panel.grid=element_blank(),

axis.line.x=element_line(size=.5, colour="black"),

axis.line.y=element_line(size=.5, colour="black"),

axis.ticks=element_line(color="black"),

axis.text=element_text(color="black", size=7),

legend.position="right",

legend.background=element_blank(),

legend.key=element_blank(),

legend.text= element_text(size=7),

text=element_text(family="sans", size=7))

p = ggplot() +

geom_line(aes(x = allerr$num, y = allerr$err.1), colour = 'grey') +

geom_line(aes(x = allerr$num, y = allerr$err.2), colour = 'grey') +

geom_line(aes(x = allerr$num, y = allerr$err.3), colour = 'grey') +

geom_line(aes(x = allerr$num, y = allerr$err.4), colour = 'grey') +

geom_line(aes(x = allerr$num, y = allerr$err.5), colour = 'grey') +

geom_line(aes(x = allerr$num, y = allerr$err.mean), colour = 'black') +

geom_vline(xintercept = optimal, colour='black', lwd=0.36, linetype="dashed") +

# geom_hline(yintercept = min(allerr$err.mean), colour='black', lwd=0.36, linetype="dashed") +

coord_trans(x = "log2") +

scale_x_continuous(breaks = c(1, 3, 5, 10, 20, 40, 60, 100, 200, 300)) + # , max(allerr$num)

labs(title=paste('Training set (n = ', dim(dat)[2],')', sep = ''),

x='Number of OTUs ', y='Cross-validation error rate') +

annotate("text", x = optimal, y = max(allerr$err.mean), label=paste("optimal = ", optimal, sep="")) +

main_theme

p

###模型评估

# 在测试集上预测

X_test <- ind.test[,-ncol(ind.test)]

y_test <- as.factor(ind.test[,ncol(ind.test)])

test_predictions <- predict(rf.train, newdata=X_test)

# 计算模型指标

confusion_matrix<-confusionMatrix(test_predictions,y_test)

accuracy<-confusion_matrix$overall["Accuracy"]

precision<-confusion_matrix$byClass["Pos Pred Value"]

recall<-confusion_matrix$byClass["Sensitivity"]

f1_score<-confusion_matrix$byClass["F1"]

#输出模型指标

print(confusion_matrix)

print(paste("Accuracy:",accuracy))

print(paste("Precision:",precision))

print(paste("Recall:",recall))

print(paste("F1 Score:",f1_score))

#绘制混淆矩阵热图

confusion_matrix_df<-as.data.frame.matrix(confusion_matrix$table)

colnames(confusion_matrix_df)<-c("CCC","BTC")

rownames(confusion_matrix_df)<-c("CCC","BTC")

draw_data<-round(confusion_matrix_df/rowSums(confusion_matrix_df),2)

draw_data$real<-rownames(draw_data)

library(reshape2)

library(data.table)

setDT(draw_data)

draw_data<-melt(draw_data)

ggplot(draw_data,aes(real,variable,fill=value))+

geom_tile()+

geom_text(aes(label=scales::percent(value)))+

scale_fill_gradient(low="#F0F0F0",high="#3575b5")+

labs(x="True labels",y="Predicted labels",title="Confusion matrix")+

theme_prism(border=T)+

theme(panel.border=element_blank(),

axis.ticks.y=element_blank(),

axis.ticks.x=element_blank(),

legend.position="none")

### 绘制ROC曲线

# 假设rf.train是您的随机森林模型，X_test是测试数据集，y_test是测试集的真实标签

test_predictions_prob <- predict(rf.train, newdata=X_test, type="prob")[,2]

roc_obj <- roc(response=y_test, predictor=test_predictions_prob)

# 绘制ROC曲线

plot(roc_obj, main="ROC Curve", col="#1c61b6", lwd=2, xlab="False Positive Rate (1-Specificity)", ylab="True Positive Rate (Sensitivity)")

# 计算AUC值

auc_value <- auc(roc_obj)

# 在图上添加AUC值，保留三位小数

text(x=0.8, y=0.2, labels=paste("AUC =", round(auc_value, 3)))

1. Zeng J, Zeng J, Lin K, et al. Development of a machine learning model to predict early recurrence for hepatocellular carcinoma after curative resection. Hepatobiliary Surg Nutr. 2022; 11(2):176-87.

2. Bohannan ZS, Coffman F, Mitrofanova A. Random survival forest model identifies novel biomarkers of event-free survival in high-risk pediatric acute lymphoblastic leukemia. Comput Struct Biotechnol J. 2022; 20:583-97.
