# Supplementary material for: Bile-Liver phenotype: Exploring the microbiota landscape in bile and intratumor of cholangiocarcinoma
Source: Comput Struct Biotechnol J. 2025 Mar 18;27:1173–86. doi: 10.1016/j.csbj.2025.03.030 (PMC11981758; doi:10.1016/j.csbj.2025.03.030)
Supplement: Supplementary file 3 — Supplementary material [file mmc3.docx]

**Supplementary Table 1** Comparison of baseline characteristics between the CCA and BBD groups in Cohort 5

| Baseline characteristics | Total cases | Group | | *P* |
| --- | --- | --- | --- | --- |
|  | N | BBD N(%) | CCA N(%) |  |
| Gender  Male  Female | 26  16 | 13 (56.5)  10 (43.5) | 13 (68.3)  6 (37.5) | 0.530 |
| Age (years)  <68  ≥68 | 16  26 | 9(39.1)  14(60.9) | 7(36.8)  12(63.2) | 0.879 |
| Hypertension  No  Yes | 23  19 | 13(56.5)  10(43.5) | 10(52.6)  9(47.4) | 0.801 |
| Diabetes  No  Yes | 36  6 | 20(87.0)  3(13.0) | 16(84.2)  3(15.8) | 1.000 |
| CHD  No  Yes | 38  4 | 21(91.3)  2(8.7) | 17(89.5)  2(10.5) | 1.000 |
| Other Cancers  No  Yes | 42  0 | 23(100.0)  0(0.0) | 19(100.0)  0(0.0) | 1.000 |
| IBD  No  Yes | 42  0 | 23(100.0)  0(0.0) | 19(100.0)  0(0.0) | 1.000 |

CCA Cholangiocarcinoma, BBD biliary benign disease, IBD inflammatory bowel disease, CHD coronary heart disease, * *P* < 0.05; ** *P* < 0.01. *P* < 0.05 was considered statistically significant

**Supplementary Table 2** Correlations of Sub-Biletypes with clinicopathological features

| Clinicopathological  features | Total cases | Sub-biletype | | *P* |
| --- | --- | --- | --- | --- |
|  | N | B1 N(%) | B2 N(%) |  |
| Group  CCA  BBD | 19  5 | 5(100.0)  0(0.0) | 14(73.7)  5(26.3) | 0.544 |
| Gender  Male  Female | 17  7 | 2(40.0)  3(60.0) | 15(78.9)  4(21.1) | 0.126 |
| Age (years)  <68  ≥68 | 10  14 | 1(20.0)  4(80.0) | 9(47.4)  10(52.6) | 0.358 |
| Size (cm)  <3  ≥3 | 8  11 | 1(20.0)  4(80.0) | 7(36.8)  7(36.8) | 0.234 |
| Histology  WD+MD  PD+UD | 7  12 | 3(60)  2(40) | 4(21.1)  10(52.6) | 0.239 |
| Distant metastasis  Absent  Present | 16  3 | 4(80.0)  1(20.0) | 12(63.2%)  2(10.5) | 0.521 |
| TNM state (AJCC)  0-II  III–IV | 6  13 | 0(0.0)  5(100.0) | 6(31.6)  8(42.1) | 0.084 |

CCA Cholangiocarcinoma, BBD biliary benign disease, WD well differentiated, MD moderately differentiated, PD poorly differentiated, UD undifferentiated, * *P* < 0.05; ** *P* < 0.01. *P* < 0.05 was considered statistically significant
